# Tipping the PARylation scale: Dysregulation of PAR signaling in Huntington and neurodegenerative diseases

**DOI:** 10.1177/18796397251372667

**Published:** 2025-09-04

**Authors:** Christina Peng, Tamara Maiuri, Ray Truant

**Affiliations:** 1 Department of Biochemistry and Biomedical Sciences, 3710 McMaster University, Hamilton, Canada

**Keywords:** Alzheimer disease, biochemistry, cell death pathways, DNA repair, metabolism, genetic modifiers

## Abstract

Poly(ADP-ribosyl)ation (PARylation), a crucial post-translational modification, is catalyzed by ADP-ribosyltransferases (ARTs) and has significant implications in various cellular processes, including DNA damage response, cell signaling, and immune processes. Aberrant PAR signaling is implicated in numerous neurodegenerative diseases, including Alzheimer, Parkinson, amyotrophic lateral sclerosis, and cerebellar ataxia, where increased PAR levels and PARP1 activity are commonly observed. However, Huntington disease exhibits a unique characteristic: reduced PAR levels and impaired PARP1 activity even in prodromal phase. This finding challenges the prevailing understanding of PAR's role in neurodegeneration and suggests that dysregulation of PAR signaling, whether through overactivation or suppression, can lead to neuronal dysfunction. Herein, we discuss how this balance may impact neurodegenerative diseases, and possible connections between PAR signaling and emerging modifiers of disease onset identified by HD genome-wide association studies (GWAS).

Poly(ADP-ribosyl)ation (PARylation) is a complex post-translational modification with critical roles in DNA damage response, cell signaling, and immune processes. It is a reversible post-translational modification in which ADP-ribose units from nicotinamide adenine dinucleotide (NAD^+^) are transferred onto target proteins. This reaction is primarily catalyzed by enzymes known as poly(ADP-ribose) polymerases (PARPs), with PARP1 being the most extensively studied member of the family. The modification involves the addition of ADP-ribose units, either singly (MAR) or as polymers (PAR), to target molecules, with the structural diversity of PAR chains—varying in length and branching—impacting cellular physiology.^1^ Emerging evidence underscores the significance of PAR structure in protein binding, enzymatic degradation, and phase separation processes, with implications for downstream signaling pathways and biomolecular condensate formation. Despite advances, the functional nuances of PAR chain diversity and its interplay with other cellular mechanisms remain areas of active investigation, promising novel therapeutic insights through targeted modulation of ADP-ribosyltransferase (ART) activities.

## Classifications and functions of ARTs

PARylation is carried out by a large family of ADP-ribosyltransferases (ARTs), also known as poly-ADP-ribose polymerases (PARPs).^2–4^ ARTs are present across all domains of life and carry crucial functions in many cellular processes including DNA repair, cellular transport, transcription regulation, and cell death.^5,6^ In humans alone, 17 ARTs have been identified to date.^4^

Based on evolution, eukaryotic ARTs can be classified into six clades (Table 1).^7^ Clade 1 consists of many PARylating proteins involved in DNA damage repair, and some also play a role in chromatin and transcription regulation.^5^ Notable examples of clade 1 ARTs include human PARPs 1–3, which possess DNA-binding domains and are activated by DNA damage.^8^ Clade 2 consists of a distinct class of ARTs only present in plants. Examples include RCD1 and SRO2, which regulate transcription and mediate stress and developmental responses.^9^ Clade 3 consists of a heterogeneous group of ARTs that varies greatly in structure and function.^7^ Human PARPs 7(TIPARP), 9, 10, and 14 are members of the clade with diverse functions including cell signaling, macrophage regulation, and maintaining genome stability.^7,10–12^ Clade 4 ARTs have a distinctive domain structure consisting of multiple ankyrin repeats followed by a sterile alpha motif.^7^ Human PARPs in the clade include tankyrase 1 and 2 (PARP5A and B) and play a role in protein degradation and cellular signaling.^13,14^ Clade 5 is characterized by the position of the catalytic domain in the middle of the protein. In humans, PARP4 (vPARP) has been associated with vaults, the largest ribonucleoprotein particles with unknown function.^15,18^ Clade 6 is characterized by its catalytic domain and most of its members have not been characterized functionally with no known functional domain at the N terminal region.^7^ Human PARP16 (ART15) however is one of the exceptions, and studies have shown its involvement with the endoplasmic reticulum and a potential role in nuclear transport.^16,17^ Given the emerging roles of defective DNA damage repair (DDR) in neurodegenerative diseases, we will focus on ARTs involved in DDR.

**Table 1.** Classification of ARTs.

Classification	Notable members	Notable function
Clade 1	PARP 1–3	DNA damage repair^8^
Clade 2	RCD1 and SRO2	Regulate transcription and mediate stress and developmental responses^9^
Clade 3	PARP 7, 9, 10, and 14	Cell signaling,^10^ macrophage regulation,^11^ and maintaining genome stability^12^
Clade 4	Tankyrase 1 and 2 (PARP5A and B)	Protein degradation^13,14^ and cellular signaling^13,14^
Clade 5	PARP4(vPARP)	Associated with vaults^15^
Clade 6	PARP16 (ART15)	Associated with the ER^16,17^ and a potential role in nuclear transport^16,17^

### PARP1 in DNA damage repair

Several ARTs are involved in DNA damage repair, and PARPs 1–3 in particular are DNA-dependent.^8^ PARP1 is the largest and most extensively studied ART of the three due to its abundance in cells and its involvement in a wide range of DNA damage repair processes.^8^ PARP1 is thought to carry out most of the PARylation in the cell and plays an important role in DNA damage repair.^19^ It rapidly recognizes single and double-stranded DNA damage breaks through its DNA binding domain and PARylates itself and other DNA damage repair proteins to recruit and activate them.^8^ PARP1 knockout mice are highly susceptible to DNA damaging agents, and PARP1 and PARP2 knockout results in embryonic lethality.^20,21^

A well-characterized involvement of ARTs in DNA damage repair is their role in single-stranded break (SSB) repair.^22,23^ PARP1 and 2 rapidly detect the presence of single-strand breaks and initiate the recruitment of XRCC1, DNA ligase III, and other proteins crucial to the repair process.^24,25^ PARP1 also recognizes apurinic/apyrimidinic sites but its exact involvement in base excision repair has been an area of debate.^23,26^

Double-stranded breaks (DSB) are repaired through two main repair pathways, homologous recombination (HR) and non-homologous end-joining (NHEJ), which further divides into alternative NHEJ and classic NHEJ.^27,28^ PARP1 plays an important role in DSB repair through the recognition of DNA damage, recruitment of the repair complex, and determination of the repair pathways.^23^ PARP1 and Ku compete for binding to exposed DNA ends at the site of damage to determine which DSB repair pathway will take place.^29–31^ During the repair process, PARP1 also recruits the MRN complex and activates ATM, an important signaling protein for DSB repair.^32,33^

An essential event for DNA damage repair that occurs concurrently with the repair process is the relaxation of chromatin to allow the repair complex to access DNA.^34^ The process is regulated by many post-translational modifications including PARylation, and PARP1 has been shown to PARylate all histone subunits and the linker histone H1.^35,36^ PARP1 also recruits other chromatin remodeling proteins such as histone PARylation factor 1, which further promotes histone PARylation.^37^ PARP1 also indirectly assists in DNA damage repair through replication fork stalling.^38^ During DNA replication, the replication fork may encounter damaged DNA. Stalling the replication process and efficient repair of the damaged DNA is crucial to prevent errors in DNA replication and maintain genome integrity. PARP1 is activated by the stalled replication forks, promotes HR at the sites of damage, and restarts DNA replication after the repair.^38^

PARP1 is cleaved by caspases, which does not affect PARP1 activity, but does implicate PARP1 in disease mechanisms.^39,40^

### PAR dysregulation in neurodegenerative disease

Neurodegenerative diseases are a broad spectrum of disorders with varying clinical presentations and underlying causes of disease.^41^ They are characterized by progressive loss of neurons and impairment of cognitive and motor functions.^41^ Although the precise disease mechanisms remain unknown, evidence suggests they are affected by factors including DNA damage, oxidative stress, mitochondrial impairment, and neuroinflammation.^42,43^

PARylation has been implicated in many of these mechanisms. As previously mentioned, PARylation plays an important role in DNA damage signaling, recruitment of repair proteins, and acts as a scaffold in the repair process.^44^ PARP1 promotes DNA damage repair caused by oxidative stress, and is involved in the removal of oxidized proteins, serving a protective function against oxidative stress.^45,46^ Alternatively, PARylation is an energy demanding process due to the consumption of NAD^+^, and its overactivation can result in severe NAD^+^ depletion, mitochondrial dysfunction, and eventual cell death.^47–50^ Aberrant PAR signaling has also been observed in multiple neurodegenerative diseases, including Alzheimer disease, Parkinson disease, amyotrophic lateral sclerosis, cerebellar ataxias, and Huntington disease,^42,51–53^ which we will summarize in turn, with emphasis placed on evidence from human samples (Table 2).

**Table 2.** Reports of human samples with dysregulated PAR.

Disease	Sample type	PAR signaling	Reference
AD	Brain	Increased PARP1 and PAR	Love et al.^54^
	Fibroblast	Increased PAR	Cecchi et al.^55^
	Lymphoblast	Increased PAR	Cecchi et al.^55^
PD	CSF	Increased PAR	Kam et al.^56^
	Fibroblast	Decreased PAR	Wang et al.^57^
ALS	Spinal cord astroglia	Increased PARP1	Kim et al.^58^
	Spinal cord motor neurons	Decreased PARP1	Kim et al.^58^
		Increased PAR	McGurk et al.^59^
	Brain	Increased PARP1	Kim et al.^60^
Ataxia, oculomotor apraxia, and axonal neuropathy	Fibroblast	Increased PAR	Hoch et al.^61^
AOA3	Lymphoblastoid	Increased PAR	Gueven et al.^62^
SCA7	Cerebellar neurons	Increased PAR	Stoyas et al.^63^
CONDSIAS	Fibroblast	Prolonged PAR response to H2O2	Danhauser et al.^64^
HD	CSF	Decreased PAR	Maiuri et al.^65^
	Fibroblast	Decreased PAR response to DNA damage	Maiuri et al.^65^
	iPSC-derived neurons	Decreased PAR	Maiuri et al.^65^

### Alzheimer disease (AD)

Aberrant PAR signaling has been observed in AD for over two decades, and subsequent studies have since provided further support for this link.^54,55,66,67^ Elevated PARP1 and PAR levels were observed in the AD brain compared to control,^54^ and higher levels of PARP1 PARylation were observed in AD fibroblasts and lymphoblasts, without changes in overall PARP1 levels.^55^ Genetic analyses have also linked the PARP1 gene to AD susceptibility,^67,68^ further highlighting the potential role for PAR in AD pathogenesis. However, increased PAR levels are only correlative with disease, which is a concern in many neurodegenerative diseases. Given that most of these diseases are late age onset with increased reactive oxygen species (ROS) loads, it is likely elevated PAR levels are due to elevated DNA damage. Indeed, in animal models, both olfactory and hearing loss in AD models are correlated with high PAR levels and low NAD^+^ levels.^69,70^

### Parkinson disease (PD)

The presynaptic neuronal protein, α-synuclein, has long been associated with PD.^71^ More recently, the activation of PARP1 was shown to exacerbate α-synuclein toxicity.^56^ Further, preformed fibrils of recombinant α-synuclein induced PARP1 activation, resulting in α-synuclein aggregates and cell death via parthanatos, a non-apoptotic mechanism involving PAR and apoptosis-inducing factor (AIF).^72^ The use of PARP inhibitors was able to rescue toxicity induced by the α-synuclein preformed fibrils.

In the context of 6-OHDA and MPTP mouse models, which use neurotoxins to induce PD-like symptoms, PARP1 knockout was protective against neuronal death.^73,74^ In the MPTP mouse model, PARP1 overactivation through oxidative stress also resulted in energy depletion, mitochondrial depolarization and AIF translocation.^75^ While the vast majority of evidence for a link between PAR and PD comes from mouse and *in vitro* studies, elevated PAR levels were found in the CSF of PD patients,^56^ and interactions between PAR and phosphorylated α-synuclein were detected in postmortem PD patient samples.^76^ As discussed below, dysregulated PAR signaling was also seen in PD patient fibroblasts.^57^ As with AD, this may just be indicative of more DNA damage but in these diseases, a threshold may be crossed that nets neuronal loss by parthanatos.

### Amyotrophic lateral sclerosis (ALS)

ALS affects motor neurons in the brain and spinal cord, which normally control voluntary movement and breathing. In one study investigating spinal cord tissue from ALS patients, Kim et al. found PARP1 immunoreactivity to be increased in astroglia, but reduced in spinal motor neurons.^58^ The same authors later investigated ALS brain tissue and found increased PARP1 expression in the neurons, subcortical glia, and macrophages of the motor cortex, as well as the parietal cortex and cerebellum, regions that are not typically affected in ALS.^60^ In contrast to the findings of Kim et al., McGurk et al.*,* found that the motor neurons in ALS spinal cord exhibited elevated PAR.^59^ This could be explained by higher PARP1 activity despite lower levels, similar to what was seen in AD patient fibroblasts and lymphoblasts.^55^ The mechanism by which elevated PAR signaling is connected to ALS remains unclear, but could be mediated through ALS-associated proteins TDP-43, FUS, and C9orf72, all of which can be regulated by PARP1 and PAR.^77–80^ Of the neurodegenerative diseases discussed here, ALS has the clearest connection to DNA damage as the result of a higher ROS load, with several known mutations in superoxide dismutase (SOD1).^81^

### Cerebellar ataxia

Cerebellar ataxia is a collection of movement disorders characterized by dysfunctions related to the cerebellum, resulting in the impaired control of limb and ocular movement, balance, and gait.^82^ Defective DNA damage repair and PARP dysregulation have been implicated in several cerebellar ataxias.^83^ In one case, a patient carrying mutations in the DNA repair gene *XRCC1* was diagnosed with symptoms of cerebellar ataxia, and the disease pathology was associated with hyperactivation of PARP1.^61^ Fibroblasts derived from the patient had increased levels of PAR, and loss of cerebellar neurons in a *Xrcc1*-defective mouse model was rescued by deletion of *Parp1*.^61^ Elevated PAR levels were also observed in lymphoblastoid cells from a patient with ataxia oculomotor apraxia type 3(AOA3), a rare inherited disorder characterized by hypersensitivity to oxidative stress and apoptosis resistance.^62^

Spinocerebellar ataxia type 7 (SCA7) is an autosomal dominant disorder characterized by progressive cerebellar degeneration and motor incoordination, and elevated levels of PAR were observed in cerebellar neurons of SCA7 patients.^63^ In SCA7 mouse models, NAD^+^ depletion was responsible for dysregulation of Sirt1, a NAD^+^ -dependent deacetylase. The authors therefore propose that excessive PARP1 activity may be responsible for the NAD^+^ depletion and neuronal demise.^63^ This mechanism could be relevant to other diseases with elevated PAR levels, as PARP1 activity can metabolically drain neurons of NAD^+^ leading to a severe stress of energy crisis.^84^ In a highly metabolically active cell population, this may in part explain the brain pathology predominant in these diseases.

Elevated PAR levels may arise not only by excessive polymerization, but also by deficient hydrolysis. Indeed, pathogenic variants of *ADPRHL2*, encoding the mono(ADP-ribosyl) hydrolase ARH3, have been found in individuals exhibiting neurodegenerative symptoms including cerebellar atrophy and progressive ataxia, a disorder now termed childhood-onset neurodegeneration, stress-induced, with variable ataxia and seizures (CONDSIAS) (see Bannister et al.^85^ and references therein). One of the earliest reports of ataxia-associated ARH3 deficiency showed a prolonged PAR response to H_2_O_2_ in patient fibroblasts.^64^

It is worth noting that some ataxias have been associated with normal PARP1 activity.^86^ This could be because the ataxia is caused by another mechanism, or, in the context of elevated DNA damage and oxidative stress,^86^ this could be an indication of *deficient* PAR signaling. As discussed below, insufficient PAR signaling may also result in neuronal death, providing an alternate pathogenic mechanism in neurodegenerative disease.

Thus, most of the accumulating evidence linking PAR dysregulation with neurodegenerative disease points to increased PARP1 levels or activity, leading to increased PAR levels (Table 2). As neurons are highly energy dependent, excessive production of PAR could be detrimental by depleting NAD^+^ stores^47^ or by blocking glycolysis via hexokinase inhibition.^87^ It could also lead to the translocation of AIF from the mitochondria to the nucleus, resulting in cell death by parthanatos.^52^ However, we propose that a *decrease* in PAR signaling could also lead to neuronal death, based on our findings in samples from people with Huntington disease.

### PAR dysregulation in Huntington disease

Although minimal, previous evidence supported a role for increased PAR signaling in Huntington disease, like other neurodegenerative diseases. A classic study has often been cited as an example of increased PARP1 in HD brain, however they measured the 85-kDa caspase-cleaved fragment (p85) of human PARP as a marker for apoptosis,^88^ which differs from active PAR-generating PARP1.^89^ Two more studies described the beneficial effects of PARP1 inhibition in the R6/2 mouse model of HD.^90,91^ In contrast, we recently found decreased PAR levels in the CSF of HD patients, both at pre-manifest and manifest stages.^65^ We further showed that the PAR response to DNA damage was impaired in HD patient-derived fibroblasts and neurons differentiated from iPSCs. At the molecular level, we found that both wild type and mutant huntingtin protein could bind PAR directly, but only the wild type protein was capable of stimulating PARP1 activity *in vitro*. This biochemical data was reinforced by direct molecular imaging using atomic force microscopy, where it was seen that pure, recombinant huntingtin/HAP40 dimer could be seen binding to -ve ends of growing PAR chains at the single molecule level. This provides a compelling potential mechanism in which the normal huntingtin protein functions to promote PARP1 activity, but this function is impaired in HD.

While these results are at odds with the previous reports in mice,^90,91^ it is possible that the R6/2 mouse model, which expresses only the first 81 amino acids of the 3144-amino acid huntingtin protein, may not reflect the normal function of full-length huntingtin in PAR biology, and therefore exhibit different phenotypes than patient-derived materials. Indeed, the defined PAR-binding motif in huntingtin is between residues 1790–1798, which demonstrates that this interaction cannot occur in a small amino-terminal sub fragment of huntingtin. PAR levels in those studies were not measured, so we do not know whether PAR levels were elevated in HD mice, nor whether they were decreased after treatment with PARP inhibitor. It is also possible that PARP1 inhibition was beneficial in mice (and would in fact be beneficial in HD patients), if the “treadmilling” activity of PARP1,^92^ by which PARP1 hydrolyzes NAD^+^ to generate free ADP-ribose, is responsible for the reduced PAR levels that we observed.^65^ Alternatively, the results could be explained by yet another mechanism, as is the case in a mouse model of spinal and bulbar muscular atrophy (SBMA), which benefited from olaparib treatment, despite having lower levels of PAR in quadricep tissue, via restoration of hexokinase activity and glycolysis.^93^

**Figure 1.**
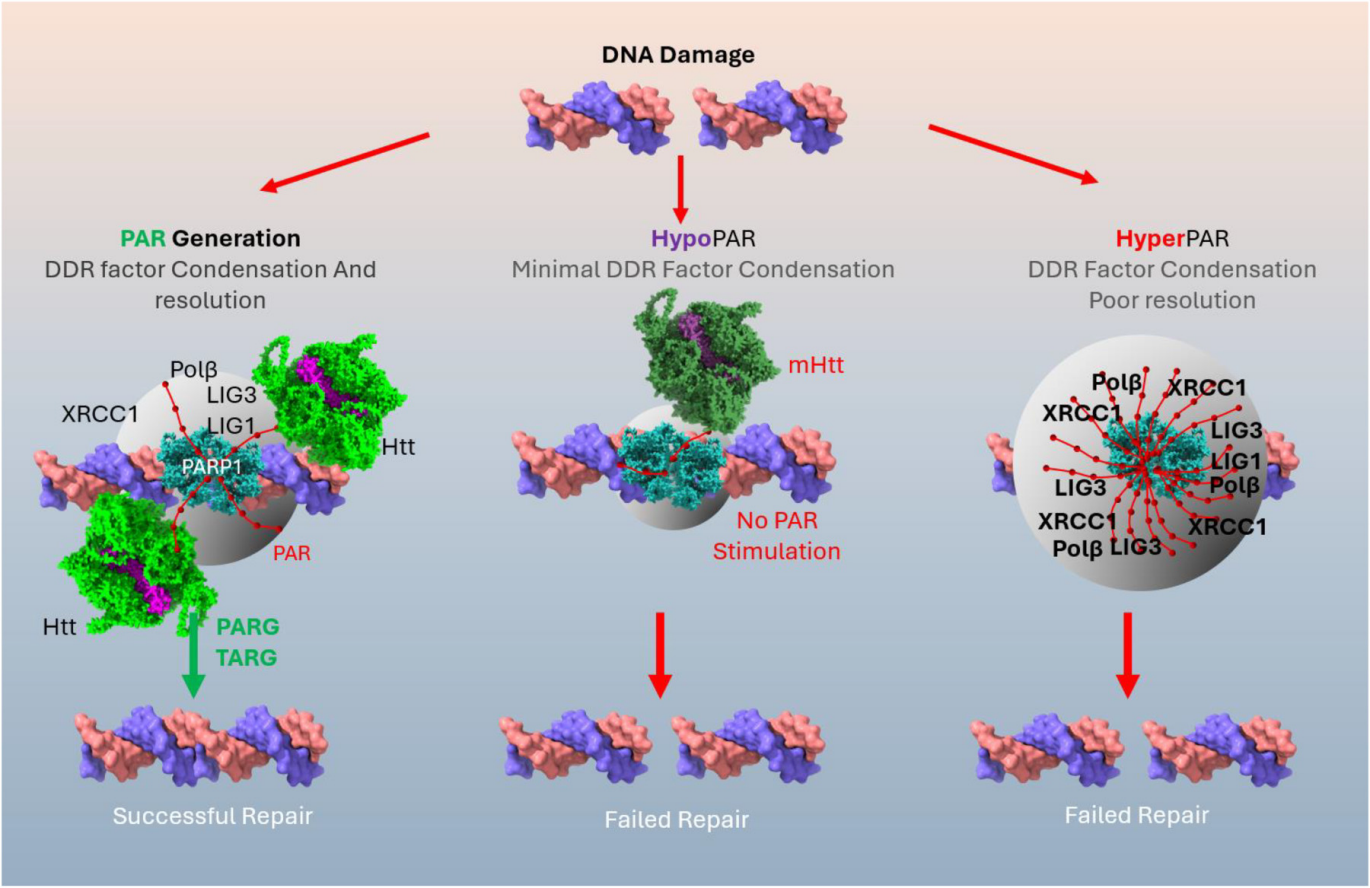
Balanced PAR signaling is required for neuronal health, while tipping the equilibrium in either direction can lead to neuronal death. PAR generation, stimulated by huntingtin, recruits DNA repair factors to DNA breaks and triggers condensation of repair factors, regulated by PAR and resolved by glycosylases PARG and TARG. HypoPARylation by failure of mutant huntingtin to stimulate PARP1 results in poor repair due to lack of proper protein recruitment. HyperPAR complexes get trapped on DNA, sequestering cell NAD^+^ levels lowering energy and resulting in failed repair.

Huntingtin is not the only neurodegenerative disease-associated protein that has been reported to promote PARP1 activity: DJ-1 is a causative gene of familial Parkinson disease (PD)^94^ that regulates DNA repair through the PARP1 pathway.^57^ Like huntingtin, the wild-type DJ-1 protein can stimulate PARP1 activity *in vitro*, and similar to our findings in HD fibroblasts, cells from PD patients with the DJ-1 mutation also have defective PARP1 activity and impaired repair of DSBs.^57^ This is in contrast to the increased PAR observed in PD CSF samples,^56^ cases which may have arisen sporadically, or by mutations in other genes. It can therefore be hypothesized that the net result of dysregulated PAR, whether increased or decreased, is a similar outcome of neuronal death (Figure 1).

Deficient PAR signaling may lead to the elevated DNA damage seen in HD models and patient samples, including the zQ175 mouse striatum^95,96^ and mouse embryo fibroblasts from the BACHD mouse,^97^ HD patient-derived fibroblasts^65,98–100^ and lymphocyte cell lines,^101^ human embryonic stem cell-derived CNS and peripheral cells^102^ and induced pluripotent cell-derived striatal neurons^95,103^ and astrocytes,^104^ peripheral blood mononuclear cells,^105,106^ and post-mortem human brain.^97^ In turn, excess DNA damage could explain mitochondrial dysfunction,^107,108^ energy depletion,^109,110^ neuroinflammation,^111,112^ dysregulated autophagy,^113,114^ and protein aggregation,^115,116^ all of which are classical observations in the field.

Recent biophysical studies on the role of PARylation and DNA repair identify a mechanism of PAR chains inducing and regulating the condensation of proteins into a liquid-liquid phase separated state.^117^ This phase separation is regulated by PAR.^118^ Biochemical and cell biology studies have identified huntingtin and polyglutamine containing huntingtin fragments as able to undergo liquid-liquid phase separation^119,120^ (Figure 1).

Given the pleiotropic role PARPs in with least 800–1000 human proteins known to be modified by PARP-mediated ADP-ribosylation. This landscape could be changed in HD and defined using quantitative mass spectrometry in cellular and *in vivo* models of HD, or from human HD brains.

### PARylation and HD GWAS

Recent genome-wide association studies (GWAS) in HD have identified DNA ligase I (LIG1) as a modifier of disease onset.^121^ Multiple proteins involved in the mismatch repair (MMR) pathway, including MSH3, FAN1, and MLH1, have also been implicated. LIG1 plays an important role in MMR through the ligation of nicked DNA caused by the repair process. LIG1 and PARP1 have complementary functions in several DNA repair pathways, which implicates LIG1 as a sensitizer of PARP1 inhibitors.^122^ Similarly, the combined dysregulation of PAR signaling and LIG1 variants in HD could further contribute to HD pathology.^122,123^

Within HD brains, LIG1 protein was seen in neurofibrillary tangles along with RRM2B or P53R2, another HD GWAS modifier,^124^ suggesting a possible mechanistic connection. While misnamed Ribonucleotide Reductase M2B, RRM2B protein is not an enzyme, it is the regulatory subunit of the ribonucleotide reductase (RNR) complex. In human disease, RRM2B mutants lead to a rare syndrome of mitochondrial DNA depletion,^125,126^ as well as a variety of rare disorders.^127^ The RNR complex can salvage ribonucleotides, such as poly-ADP ribose from transient PAR chains in conjunction with thioredoxin scaffolded on RRM2B.^128^ The thioredoxin pathway is a fundamental cellular system responsible for maintaining redox homeostasis by facilitating the reduction of disulfide bonds in proteins. As with PARylation, thioredoxin activity is fuelled by NAD^+^ . Thus with LIG1 and RRM2B, there is a crossover of regulation between cell metabolism, NAD^+^ levels and PARylation. As a synthetic lethal in *BRCA1* breast cancer tumors, LIG1 inactivation results in increased PARylation.^129^

Like huntingtin, RRM2B is regulated by TP53.^130^ Ergo, any cell based studies of RRM2B need to avoid transformed cell lines in which TP53 pathways are typically inhibited.^131^ Deficiency in TP53/RRM2B results in the activation of an NRF2 antioxidant transcriptional program, with a concomitant elevation in basal PARylation in cells.^131^ As a corollary, RRM2B suppresses activation of the oxidative stress pathway and is up-regulated by TP53 during senescence.^132^ The effect of NRF2 on elevating the PAR response may have therapeutic implications in Huntington disease with modern NRF2 stabilizing drugs like omaveloxolone (Skyclarys), recently approved for Friedreich's ataxia.^133^

### Outlook

Here, we have summarized the reports of dysregulated PAR signaling in human neurodegenerative disease samples. While many conditions, such as Alzheimer, Parkinson, ALS, and ataxia, are predominantly characterized by elevated PAR levels and PARP1 overactivation, Huntington disease presents a striking exception, with reduced PAR levels and impaired PARP1 activity. In the human biology context, Huntington disease gene carriers have a significantly reduced incidence of some cancer types, consistent with the success of PARP1 inhibitor drugs in oncology applications. It could be important to know outcomes of any HD patients treated at any time in their lives with PARP1 inhibitors and how this affected cancer treatment or had any effect on HD symptoms. The widespread ethnic incidence of HD across the world^134^ suggests that a mutant huntingtin gene may have conferred a genetic advantage in evolution to avoid early life cancers and thus a better chance of procreation.

We present a model in which balanced PAR signaling is required for neuronal health, while tipping the equilibrium in either direction can lead to neuronal death. This diversity in PAR dysregulation highlights the need to consider disease-specific profiles of PAR signaling when developing therapeutic interventions. Moving forward, it will be crucial to explore both PARP inhibitors and PARG inhibitors as potential treatments, tailoring their use to the unique signaling patterns observed in each disorder. Broader efforts to identify cases of reduced PAR signaling and elucidate their underlying mechanisms will provide valuable insights, paving the way for novel therapeutic strategies and a more nuanced understanding of PAR biology in neurodegeneration.

## References

[1] Reber JM Mangerich A . Why structure and chain length matter: on the biological significance underlying the structural heterogeneity of poly(ADP-ribose). Nucleic Acids Res 2021; 49: 8432–8448. 34302489 10.1093/nar/gkab618 PMC8421145

[2] Thomas C Tulin AV . Poly-ADP-ribose polymerase: machinery for nuclear processes. Mol Aspects Med 2013; 34: 1124–1137. 23624145 10.1016/j.mam.2013.04.001 PMC3750069

[3] Liu C Yu X . ADP-ribosyltransferases and poly ADP-ribosylation. Curr Protein Pept Sci 2015; 16: 491–501. 25938242 10.2174/1389203716666150504122435 PMC4725697

[4] Lüscher B Ahel I Altmeyer M , et al. ADP-ribosyltransferases, an update on function and nomenclature. FEBS J 2022; 289: 7399–7410. 34323016 10.1111/febs.16142 PMC9027952

[5] Perina D Mikoč A Ahel J , et al. Distribution of protein poly(ADP-ribosyl)ation systems across all domains of life. DNA Repair (Amst) 2014; 23: 4–16. 24865146 10.1016/j.dnarep.2014.05.003 PMC4245714

[6] Hassa PO Hottiger MO . The diverse biological roles of mammalian PARPS, a small but powerful family of poly-ADP-ribose polymerases. Front Biosci 2008; 13: 3046–3082. 17981777 10.2741/2909

[7] Citarelli M Teotia S Lamb RS . Evolutionary history of the poly(ADP-ribose) polymerase gene family in eukaryotes. BMC Evol Biol 2010; 10: 308. 20942953 10.1186/1471-2148-10-308 PMC2964712

[8] van Beek L McClay É Patel S , et al. PARP Power: a structural perspective on PARP1, PARP2, and PARP3 in DNA damage repair and nucleosome remodelling. Int J Mol Sci 2021; 22: 5112. 34066057 10.3390/ijms22105112 PMC8150716

[9] Jaspers P Blomster T Brosché M , et al. Unequally redundant RCD1 and SRO1 mediate stress and developmental responses and interact with transcription factors. Plant J 2009; 60: 268–279. 19548978 10.1111/j.1365-313X.2009.03951.x

[10] Yamada T Horimoto H Kameyama T , et al. Constitutive aryl hydrocarbon receptor signaling constrains type I interferon-mediated antiviral innate defense. Nat Immunol 2016; 17: 687–694. 27089381 10.1038/ni.3422

[11] Iwata H Goettsch C Sharma A , et al. PARP9 And PARP14 cross-regulate macrophage activation via STAT1 ADP-ribosylation. Nat Commun 2016; 7: 12849. 27796300 10.1038/ncomms12849 PMC5095532

[12] Nicolae CM Aho ER Vlahos AHS , et al. The ADP-ribosyltransferase PARP10/ARTD10 interacts with proliferating cell nuclear antigen (PCNA) and is required for DNA damage tolerance. J Biol Chem 2014; 289: 13627–13637. 24695737 10.1074/jbc.M114.556340 PMC4036367

[13] Vivelo CA Ayyappan V Leung AKL . Poly(ADP-ribose)-dependent ubiquitination and its clinical implications. Biochem Pharmacol 2019; 167: 3–12. 31077644 10.1016/j.bcp.2019.05.006 PMC6702056

[14] Yang E Tacchelly-Benites O Wang Z , et al. Wnt pathway activation by ADP-ribosylation. Nat Commun 2016; 7: 11430. 27138857 10.1038/ncomms11430 PMC4857404

[15] Kickhoefer VA Siva AC Kedersha NL , et al. The 193-kD vault protein, VPARP, is a novel poly(ADP-ribose) polymerase. J Cell Biol 1999; 146: 917–928. 10477748 10.1083/jcb.146.5.917 PMC2169495

[16] Di Paola S Micaroni M Di Tullio G , et al. PARP16/ARTD15 Is a novel endoplasmic-reticulum-associated mono-ADP-ribosyltransferase that interacts with, and modifies karyopherin-ß1. PLoS One 2012; 7: e37352. 10.1371/journal.pone.0037352 PMC3372510 22701565

[17] Jwa M Chang P . PARP16 Is a tail-anchored endoplasmic reticulum protein required for the PERK- and IRE1α-mediated unfolded protein response. Nat Cell Biol 2012; 14: 1223–1230. 23103912 10.1038/ncb2593 PMC3494284

[18] Berger W Steiner E Grusch M , et al. Vaults and the major vault protein: novel roles in signal pathway regulation and immunity. Cell Mol Life Sci 2009; 66: 43–61. 18759128 10.1007/s00018-008-8364-z PMC11131553

[19] Kamaletdinova T Fanaei-Kahrani Z Wang Z-Q . The enigmatic function of PARP1: from PARylation activity to PAR readers. Cells 2019; 8: 1625. 31842403 10.3390/cells8121625 PMC6953017

[20] Shibata A Kamada N Masumura K-I , et al. Parp-1 deficiency causes an increase of deletion mutations and insertions/rearrangements in vivo after treatment with an alkylating agent. Oncogene 2005; 24: 1328–1337. 15608683 10.1038/sj.onc.1208289

[21] Ménissier de Murcia J Ricoul M Tartier L , et al. Functional interaction between PARP-1 and PARP-2 in chromosome stability and embryonic development in mouse. EMBO J 2003; 22: 2255–2263. 12727891 10.1093/emboj/cdg206 PMC156078

[22] Satoh MS Lindahl T . Role of poly(ADP-ribose) formation in DNA repair. Nature 1992; 356: 356–358. 1549180 10.1038/356356a0

[23] Ray Chaudhuri A Nussenzweig A . The multifaceted roles of PARP1 in DNA repair and chromatin remodelling. Nat Rev Mol Cell Biol 2017; 18: 610–621. 28676700 10.1038/nrm.2017.53 PMC6591728

[24] Hanzlikova H Gittens W Krejcikova K , et al. Overlapping roles for PARP1 and PARP2 in the recruitment of endogenous XRCC1 and PNKP into oxidized chromatin. Nucleic Acids Res 2017; 45: 2546–2557. 27965414 10.1093/nar/gkw1246 PMC5389470

[25] El-Khamisy SF Masutani M Suzuki H , et al. A requirement for PARP-1 for the assembly or stability of XRCC1 nuclear foci at sites of oxidative DNA damage. Nucleic Acids Res 2003; 31: 5526–5533. 14500814 10.1093/nar/gkg761 PMC206461

[26] Das BB Huang S-YN Murai J , et al. PARP1-TDP1 Coupling for the repair of topoisomerase I-induced DNA damage. Nucleic Acids Res 2014; 42: 4435–4449. 24493735 10.1093/nar/gku088 PMC3985661

[27] Deriano L Roth DB . Modernizing the nonhomologous end-joining repertoire: alternative and classical NHEJ share the stage. Annu Rev Genet 2013; 47: 433–455. 24050180 10.1146/annurev-genet-110711-155540

[28] O’Driscoll M Jeggo PA . The role of double-strand break repair - insights from human genetics. Nat Rev Genet 2006; 7: 45–54. 16369571 10.1038/nrg1746

[29] Wang M Wu W Wu W , et al. PARP-1 and Ku compete for repair of DNA double strand breaks by distinct NHEJ pathways. Nucleic Acids Res 2006; 34: 6170–6182. 17088286 10.1093/nar/gkl840 PMC1693894

[30] Xie S Mortusewicz O Ma HT , et al. Timeless interacts with PARP-1 to promote homologous recombination repair. Mol Cell 2015; 60: 163–176. 26344098 10.1016/j.molcel.2015.07.031

[31] Hochegger H Dejsuphong D Fukushima T , et al. Parp-1 protects homologous recombination from interference by Ku and Ligase IV in vertebrate cells. EMBO J 2006; 25: 1305–1314. 16498404 10.1038/sj.emboj.7601015 PMC1422167

[32] Aguilar-Quesada R Muñoz-Gámez JA Martín-Oliva D , et al. Interaction between ATM and PARP-1 in response to DNA damage and sensitization of ATM deficient cells through PARP inhibition. BMC Mol Biol 2007; 8: 29. 17459151 10.1186/1471-2199-8-29 PMC1868035

[33] Ciccia A Elledge SJ . The DNA damage response: making it safe to play with knives. Mol Cell 2010; 40: 179–204. 20965415 10.1016/j.molcel.2010.09.019 PMC2988877

[34] Zong W Gong Y Sun W , et al. PARP1: Liaison of chromatin remodeling and transcription. Cancers (Basel) 2022; 14: 4162. 36077699 10.3390/cancers14174162 PMC9454564

[35] Li Z Li Y Tang M , et al. Destabilization of linker histone H1.2 is essential for ATM activation and DNA damage repair. Cell Res 2018; 28: 756–770. 29844578 10.1038/s41422-018-0048-0 PMC6028381

[36] Messner S Altmeyer M Zhao H , et al. PARP1 ADP-ribosylates lysine residues of the core histone tails. Nucleic Acids Res 2010; 38: 6350–6362. 20525793 10.1093/nar/gkq463 PMC2965223

[37] Gibbs-Seymour I Fontana P Rack JGM , et al. HPF1/C4orf27 Is a PARP-1-interacting protein that regulates PARP-1 ADP-ribosylation activity. Mol Cell 2016; 62: 432–442. 27067600 10.1016/j.molcel.2016.03.008 PMC4858568

[38] Bryant HE Petermann E Schultz N , et al. PARP Is activated at stalled forks to mediate Mre11-dependent replication restart and recombination. EMBO J 2009; 28: 2601–2615. 19629035 10.1038/emboj.2009.206 PMC2738702

[39] Ruiz C Casarejos MJ Gomez A , et al. Protection by glia-conditioned medium in a cell model of Huntington disease. PLoS Curr 2012; 4: e4fbca54a2028b. 10.1371/4fbca54a2028b PMC3423315 22919565

[40] Paldino E D’Angelo V Laurenti D , et al. Modulation of inflammasome and pyroptosis by Olaparib, a PARP-1 inhibitor, in the R6/2 mouse model of Huntington’s disease. Cells 2020; 9: 2286. 33066292 10.3390/cells9102286 PMC7602058

[41] Przedborski S Vila M Jackson-Lewis V . Neurodegeneration: what is it and where are we? J Clin Invest 2003; 111: 3–10. 12511579 10.1172/JCI17522 PMC151843

[42] Hu M-L Pan Y-R Yong Y-Y , et al. Poly (ADP-ribose) polymerase 1 and neurodegenerative diseases: past, present, and future. Ageing Res Rev 2023; 91: 102078. 37758006 10.1016/j.arr.2023.102078

[43] Shadfar S Parakh S Jamali MS , et al. Redox dysregulation as a driver for DNA damage and its relationship to neurodegenerative diseases. Transl Neurodegener 2023; 12: 1–34. 37055865 10.1186/s40035-023-00350-4 PMC10103468

[44] Pascal JM . The comings and goings of PARP-1 in response to DNA damage. DNA Repair (Amst) 2018; 71: 177–182. 30177435 10.1016/j.dnarep.2018.08.022 PMC6637744

[45] Luo X Kraus WL . On PAR with PARP: cellular stress signaling through poly(ADP-ribose) and PARP-1. Genes Dev 2012; 26: 417–432. 22391446 10.1101/gad.183509.111 PMC3305980

[46] Bakondi E Catalgol B Bak I , et al. Age-related loss of stress-induced nuclear proteasome activation is due to low PARP-1 activity. Free Radic Biol Med 2011; 50: 86–92. 20977936 10.1016/j.freeradbiomed.2010.10.700

[47] Alano CC Garnier P Ying W , et al. NAD+ depletion is necessary and sufficient for poly(ADP-ribose) polymerase-1-mediated neuronal death. J Neurosci 2010; 30: 2967–2978. 20181594 10.1523/JNEUROSCI.5552-09.2010 PMC2864043

[48] Andrabi SA Dawson TM Dawson VL . Mitochondrial and nuclear cross talk in cell death: parthanatos: parthanatos. Ann N Y Acad Sci 2008; 1147: 233–241. 19076445 10.1196/annals.1427.014 PMC4454457

[49] Los M Mozoluk M Ferrari D , et al. Activation and caspase-mediated inhibition of PARP: a molecular switch between fibroblast necrosis and apoptosis in death receptor signaling. Mol Biol Cell 2002; 13: 978–988. 11907276 10.1091/mbc.01-05-0272 PMC99613

[50] Ha HC Snyder SH . Poly(ADP-ribose) polymerase is a mediator of necrotic cell death by ATP depletion. Proc Natl Acad Sci U S A 1999; 96: 13978–13982. 10570184 10.1073/pnas.96.24.13978 PMC24176

[51] Mao K Zhang G . The role of PARP1 in neurodegenerative diseases and aging. FEBS J 2022; 289: 2013–2024. 33460497 10.1111/febs.15716

[52] Park H Kam T-I Dawson TM , et al. Poly (ADP-ribose) (PAR)-dependent cell death in neurodegenerative diseases. Int Rev Cell Mol Biol 2020; 353: 1–29. 32381174 10.1016/bs.ircmb.2019.12.009

[53] Thapa K Khan H Sharma U , et al. Poly (ADP-ribose) polymerase-1 as a promising drug target for neurodegenerative diseases. Life Sci 2021; 267: 118975. 33387580 10.1016/j.lfs.2020.118975

[54] Love S Barber R Wilcock GK . Increased poly(ADP-ribosyl)ation of nuclear proteins in Alzheimer’s disease. Brain 1999; 122: 247–253. 10071053 10.1093/brain/122.2.247

[55] Cecchi C Fiorillo C Sorbi S , et al. Oxidative stress and reduced antioxidant defenses in peripheral cells from familial Alzheimer’s patients. Free Radic Biol Med 2002; 33: 1372–1379. 12419469 10.1016/s0891-5849(02)01049-3

[56] Kam T-I Mao X Park H , et al. Poly(ADP-ribose) drives pathologic α-synuclein neurodegeneration in Parkinson’s disease. Science 2018; 362: eaat8407. 10.1126/science.aat8407 PMC6431793 30385548

[57] Wang Z-X Liu Y Li Y-L , et al. Nuclear DJ-1 regulates DNA damage repair via the regulation of PARP1 activity. Int J Mol Sci 2023; 24: 8651. 37239999 10.3390/ijms24108651 PMC10218208

[58] Kim SH Henkel JS Beers DR , et al. PARP Expression is increased in astrocytes but decreased in motor neurons in the spinal cord of sporadic ALS patients. J Neuropathol Exp Neurol 2003; 62: 88–103. 12528821 10.1093/jnen/62.1.88

[59] McGurk L Mojsilovic-Petrovic J Van Deerlin VM , et al. Nuclear poly(ADP-ribose) activity is a therapeutic target in amyotrophic lateral sclerosis. Acta Neuropathol Commun 2018; 6: 84. 30157956 10.1186/s40478-018-0586-1 PMC6114235

[60] Kim SH Engelhardt JI Henkel JS , et al. Widespread increased expression of the DNA repair enzyme PARP in brain in ALS. Neurology 2004; 62: 319–322. 14745081 10.1212/01.wnl.0000103291.04985.dc

[61] Hoch NC Hanzlikova H Rulten SL , et al. XRCC1 Mutation is associated with PARP1 hyperactivation and cerebellar ataxia. Nature 2017; 541: 87–91. 28002403 10.1038/nature20790 PMC5218588

[62] Gueven N Becherel OJ Howe O , et al. A novel form of ataxia oculomotor apraxia characterized by oxidative stress and apoptosis resistance. Cell Death Differ 2007; 14: 1149–1161. 17347666 10.1038/sj.cdd.4402116

[63] Stoyas CA Bushart DD Switonski PM , et al. Nicotinamide pathway-dependent Sirt1 activation restores calcium homeostasis to achieve neuroprotection in spinocerebellar ataxia type 7. Neuron 2020; 105: 630–644.e9. 31859031 10.1016/j.neuron.2019.11.019 PMC7147995

[64] Danhauser K Alhaddad B Makowski C , et al. Bi-allelic ADPRHL2 mutations cause neurodegeneration with developmental delay, ataxia, and axonal neuropathy. Am J Hum Genet 2018; 103: 817–825. 30401461 10.1016/j.ajhg.2018.10.005 PMC6218634

[65] Maiuri T Bazan CB Harding RJ , et al. Poly ADP-ribose signaling is dysregulated in Huntington disease. Proc Natl Acad Sci U S A 2024; 121: e2318098121. 10.1073/pnas.2318098121 PMC11459172 39331414

[66] Martire S Fuso A Rotili D , et al. PARP-1 modulates amyloid beta peptide-induced neuronal damage. PLoS One 2013; 8: e72169. 10.1371/journal.pone.0072169 PMC3782458 24086258

[67] Liu H-P Lin W-Y Wu B-T , et al. Evaluation of the poly(ADP-ribose) polymerase-1 gene variants in Alzheimer’s disease. J Clin Lab Anal 2010; 24: 182–186. 20486200 10.1002/jcla.20379 PMC6647671

[68] Infante J Llorca J Mateo I , et al. Interaction between poly(ADP-ribose) polymerase 1 and interleukin 1A genes is associated with Alzheimer’s disease risk. Dement Geriatr Cogn Disord 2007; 23: 215–218. 17290104 10.1159/000099471

[69] Dan X Yang B McDevitt RA , et al. Loss of smelling is an early marker of aging and is associated with inflammation and DNA damage in C57BL/6J mice. Aging Cell 2023; 22: e13793. 10.1111/acel.13793 PMC10086518 36846960

[70] Park J-H Sahbaz BD Pekhale K , et al. Early-Onset hearing loss in mouse models of Alzheimer’s disease and increased DNA damage in the cochlea. Aging Biol 2024; 1: 20240025. 38500536 10.59368/agingbio.20240025 PMC10948084

[71] Calabresi P Mechelli A Natale G , et al. Alpha-synuclein in Parkinson’s disease and other synucleinopathies: from overt neurodegeneration back to early synaptic dysfunction. Cell Death Dis 2023; 14: 1–16. 36859484 10.1038/s41419-023-05672-9 PMC9977911

[72] Yang L Guttman L Dawson VL , et al. Parthanatos: mechanisms, modulation, and therapeutic prospects in neurodegenerative disease and stroke. Biochem Pharmacol 2024; 228: 116174. 38552851 10.1016/j.bcp.2024.116174 PMC11410548

[73] Kim TW Cho HM Choi SY , et al. (ADP-ribose) polymerase 1 and AMP-activated protein kinase mediate progressive dopaminergic neuronal degeneration in a mouse model of Parkinson’s disease. Cell Death Dis 2013; 4: e919. 10.1038/cddis.2013.447 PMC3847323 24232095

[74] Mandir AS Przedborski S Jackson-Lewis V , et al. Poly(ADP-ribose) polymerase activation mediates 1-methyl-4-phenyl-1, 2,3,6-tetrahydropyridine (MPTP)-induced parkinsonism. Proc Natl Acad Sci U S A 1999; 96: 5774–5779. 10318960 10.1073/pnas.96.10.5774 PMC21936

[75] Yu C Kim B-S Kim E . FAF1 Mediates regulated necrosis through PARP1 activation upon oxidative stress leading to dopaminergic neurodegeneration. Cell Death Differ 2016; 23: 1873–1885. 27662363 10.1038/cdd.2016.99 PMC5071579

[76] Puentes LN Lengyel-Zhand Z Lee JY , et al. Poly (ADP-ribose) interacts with phosphorylated α-synuclein in post mortem PD samples. Front Aging Neurosci 2021; 13: 704041. 34220490 10.3389/fnagi.2021.704041 PMC8249773

[77] McGurk L Rifai OM Bonini NM . Poly(ADP-ribosylation) in age-related neurological disease. Trends Genet 2019; 35: 601–613. 31182245 10.1016/j.tig.2019.05.004 PMC6625889

[78] Gao J Mewborne QT Girdhar A , et al. Poly(ADP-ribose) promotes toxicity of C9ORF72 arginine-rich dipeptide repeat proteins. Sci Transl Med 2022; 14: eabq3215. 10.1126/scitranslmed.abq3215 PMC10359073 36103513

[79] Rhine K Dasovich M Yoniles J , et al. Poly(ADP-ribose) drives condensation of FUS via a transient interaction. Mol Cell 2022; 82: 969–985.e11. 35182479 10.1016/j.molcel.2022.01.018 PMC9330637

[80] Khodyreva SN Dyrkheeva NS Lavrik OI . Proteins associated with neurodegenerative diseases: link to DNA repair. Biomedicines 2024; 12: 2808. 39767715 10.3390/biomedicines12122808 PMC11673744

[81] Kim G Gautier O Tassoni-Tsuchida E , et al. ALS Genetics: gains, losses, and implications for future therapies. Neuron 2020; 108: 822–842. 32931756 10.1016/j.neuron.2020.08.022 PMC7736125

[82] Marsden JF . Cerebellar ataxia. Handb Clin Neurol 2018; 159: 261–281. 30482319 10.1016/B978-0-444-63916-5.00017-3

[83] Coon EA Benarroch EE . DNA Damage response: selected review and neurologic implications. Neurology 2018; 90: 367–376. 29352098 10.1212/WNL.0000000000004989 PMC10681070

[84] Formentini L Macchiarulo A Cipriani G , et al. Poly(ADP-ribose) catabolism triggers AMP-dependent mitochondrial energy failure. J Biol Chem 2009; 284: 17668–17676. 19411252 10.1074/jbc.M109.002931 PMC2719406

[85] Bannister M Bray S Aggarwal A , et al. An ADPRS variant disrupts ARH3 stability and subcellular localization in children with neurodegeneration and respiratory failure. HGG Adv 2025; 6: 100386. 39580621 10.1016/j.xhgg.2024.100386 PMC11667697

[86] Lavin MF Gueven N Grattan-Smith P . Defective responses to DNA single- and double-strand breaks in spinocerebellar ataxia. DNA Repair (Amst) 2008; 7: 1061–1076. 18467193 10.1016/j.dnarep.2008.03.008

[87] Andrabi SA Umanah GK Chang C , et al. Poly(ADP-ribose) polymerase-dependent energy depletion occurs through inhibition of glycolysis. Proc Natl Acad Sci U S A 2014; 111: 10209–10214. 24987120 10.1073/pnas.1405158111 PMC4104885

[88] Vis JC Schipper E de Boer-van Huizen RT , et al. Expression pattern of apoptosis-related markers in Huntington’s disease. Acta Neuropathol 2005; 109: 321–328. 15668790 10.1007/s00401-004-0957-5

[89] Mashimo M Onishi M Uno A , et al. The 89-kDa PARP1 cleavage fragment serves as a cytoplasmic PAR carrier to induce AIF-mediated apoptosis. J Biol Chem 2021; 296: 100046. 33168626 10.1074/jbc.RA120.014479 PMC7948984

[90] Cardinale A Paldino E Giampà C , et al. PARP-1 Inhibition is neuroprotective in the R6/2 mouse model of Huntington’s disease. PLoS One 2015; 10: e0134482. 10.1371/journal.pone.0134482 PMC4529170 26252217

[91] Paldino E Cardinale A D’Angelo V , et al. Selective sparing of striatal interneurons after poly (ADP-ribose) polymerase 1 inhibition in the R6/2 mouse model of Huntington’s disease. Front Neuroanat 2017; 11: 61. 28824383 10.3389/fnana.2017.00061 PMC5539174

[92] Rudolph J Roberts G Muthurajan UM , et al. HPF1 and nucleosomes mediate a dramatic switch in activity of PARP1 from polymerase to hydrolase. Elife 2021; 10: e65773. 10.7554/eLife.65773 PMC8012059 33683197

[93] Garcia Castro DR Mazuk JR Heine EM , et al. Increased SIRT3 combined with PARP inhibition rescues motor function of SBMA mice. iScience 2023; 26: 107375. 37599829 10.1016/j.isci.2023.107375 PMC10433013

[94] Dolgacheva LP Berezhnov AV Fedotova EI , et al. Role of DJ-1 in the mechanism of pathogenesis of Parkinson’s disease. J Bioenerg Biomembr 2019; 51: 175–188. 31054074 10.1007/s10863-019-09798-4 PMC6531411

[95] Gao R Chakraborty A Geater C , et al. Mutant huntingtin impairs PNKP and ATXN3, disrupting DNA repair and transcription. eLife 2019; 8: e42988. 10.7554/eLife.42988 PMC6529219 30994454

[96] Morozko EL Smith-Geater C Monteys AM , et al. PIAS1 Modulates striatal transcription, DNA damage repair, and SUMOylation with relevance to Huntington’s disease. Proc Natl Acad Sci U S A 2021; 118: e2021836118. 10.1073/pnas.2021836118 PMC7848703 33468657

[97] Lu X-H Mattis VB Wang N , et al. Targeting ATM ameliorates mutant huntingtin toxicity in cell and animal models of huntington’s disease. Sci Transl Med 2014; 6: 268ra178. 10.1126/scitranslmed.3010523 25540325

[98] Maiuri T Mocle AJ Hung CL , et al. Huntingtin is a scaffolding protein in the ATM oxidative DNA damage response complex. Hum Mol Genet 2017; 26: 395–406. 28017939 10.1093/hmg/ddw395

[99] Scudiero DA Meyer SA Clatterbuck BE , et al. Hypersensitivity to N-methyl-n’-nitro-N-nitrosoguanidine in fibroblasts from patients with Huntington disease, familial dysautonomia, and other primary neuronal degenerations. Proc Natl Acad Sci U S A 1981; 78: 6451–6455. 6458814 10.1073/pnas.78.10.6451 PMC349057

[100] Ferlazzo ML Sonzogni L Granzotto A , et al. Mutations of the Huntington’s disease protein impact on the ATM-dependent signaling and repair pathways of the radiation-induced DNA double-strand breaks: corrective effect of statins and bisphosphonates. Mol Neurobiol 2014; 49: 1200–1211. 24277524 10.1007/s12035-013-8591-7

[101] Moshell AN Tarone RE Barrett SF , et al. Radiosensitivity in Huntington’s disease: implications for pathogenesis and presymptomatic diagnosis. Lancet 1980; 1: 9–11. 6101401 10.1016/s0140-6736(80)90550-4

[102] Ooi J Langley SR Xu X , et al. Unbiased profiling of isogenic Huntington disease hPSC-derived CNS and peripheral cells reveals strong cell-type specificity of CAG length effects. Cell Rep 2019; 26: 2494–2508.e7. 30811996 10.1016/j.celrep.2019.02.008

[103] Palminha NM Dos Santos Souza C Griffin J , et al. Defective repair of topoisomerase I induced chromosomal damage in Huntington’s disease. Cell Mol Life Sci 2022; 79: 160. 35224690 10.1007/s00018-022-04204-6 PMC8882575

[104] Lange J Gillham O Flower M , et al. Polyq length-dependent metabolic alterations and DNA damage drive human astrocyte dysfunction in Huntington’s disease. Prog Neurobiol 2023; 225: 102448. 37023937 10.1016/j.pneurobio.2023.102448

[105] Askeland G Dosoudilova Z Rodinova M , et al. Increased nuclear DNA damage precedes mitochondrial dysfunction in peripheral blood mononuclear cells from huntington’s disease patients. Sci Rep 2018; 8: 9817. 29959348 10.1038/s41598-018-27985-y PMC6026140

[106] Castaldo I De Rosa M Romano A , et al. DNA Damage signatures in peripheral blood cells as biomarkers in prodromal huntington disease. Ann Neurol 2019; 85: 296–301. 30549309 10.1002/ana.25393

[107] Patel J Baptiste BA Kim E , et al. DNA Damage and mitochondria in cancer and aging. Carcinogenesis 2020; 41: 1625–1634. 33146705 10.1093/carcin/bgaa114 PMC7791626

[108] Fang EF Scheibye-Knudsen M Chua KF , et al. Nuclear DNA damage signalling to mitochondria in ageing. Nat Rev Mol Cell Biol 2016; 17: 308–321. 26956196 10.1038/nrm.2016.14 PMC5161407

[109] Ruszkiewicz JA Bürkle A Mangerich A . Fueling genome maintenance: on the versatile roles of NAD+ in preserving DNA integrity. J Biol Chem 2022; 298: 102037. 35595095 10.1016/j.jbc.2022.102037 PMC9194868

[110] Morales JC Li L Fattah FJ , et al. Review of poly (ADP-ribose) polymerase (PARP) mechanisms of action and rationale for targeting in cancer and other diseases. Crit Rev Eukaryot Gene Expr 2014; 24: 15–28. 24579667 10.1615/critreveukaryotgeneexpr.2013006875 PMC4806654

[111] Zhang W Sun H-S Wang X , et al. Cellular senescence, DNA damage, and neuroinflammation in the aging brain. Trends Neurosci 2024; 47: 461–474. 38729785 10.1016/j.tins.2024.04.003

[112] Song X Ma F Herrup K . DNA damage-induced neuroinflammation in neurodegenerative disease. Alzheimer’s Dement 2022; 17: e055175.

[113] Juretschke T Beli P . Causes and consequences of DNA damage-induced autophagy. Matrix Biol 2021; 100-101: 39–53. 33600946 10.1016/j.matbio.2021.02.004

[114] Demi̇rbağ-Sarikaya S Çakir H Gözüaçik D , et al. Crosstalk between autophagy and DNA repair systems. Turk J Biol 2021; 45: 35. 34377049 10.3906/biy-2103-51 PMC8313936

[115] Huiting W Dekker SL van der Lienden JCJ , et al. Targeting DNA topoisomerases or checkpoint kinases results in an overload of chaperone systems, triggering aggregation of a metastable subproteome. Elife 2022; 11: e70726. 10.7554/eLife.70726 PMC8871389 35200138

[116] Paull TT . DNA Damage and regulation of protein homeostasis. DNA Repair 2021; 105: 103155. 34116476 10.1016/j.dnarep.2021.103155 PMC8364502

[117] Chin Sang C Moore G Tereshchenko M , et al. PARP1 Condensates differentially partition DNA repair proteins and enhance DNA ligation. EMBO Rep 2024; 25: 5635–5666. 39496836 10.1038/s44319-024-00285-5 PMC11624282

[118] Rhine K Odeh HM Shorter J , et al. Regulation of biomolecular condensates by poly(ADP-ribose). Chem Rev 2023; 123: 9065–9093. 37115110 10.1021/acs.chemrev.2c00851 PMC10524010

[119] Yang J Yang X . Phase transition of huntingtin: factors and pathological relevance. Front Genet 2020; 11: 754. 32849783 10.3389/fgene.2020.00754 PMC7396480

[120] Pandey NK Varkey J Ajayan A , et al. Fluorescent protein tagging promotes phase separation and alters the aggregation pathway of huntingtin exon-1. J Biol Chem 2024; 300: 105585. 38141760 10.1016/j.jbc.2023.105585 PMC10825056

[121] Lee J-M Huang Y Orth M , et al. Genetic modifiers of huntington disease differentially influence motor and cognitive domains. Am J Hum Genet 2022; 109: 885–899. 35325614 10.1016/j.ajhg.2022.03.004 PMC9118113

[122] Fracassi G Lorenzin F Orlando F , et al. CRISPR/Cas9 screens identify LIG1 as a sensitizer of PARP inhibitors in castration-resistant prostate cancer. J Clin Invest 2024; 135: e179393. 10.1172/JCI179393 PMC11827843 39718835

[123] Li G-M . New insights and challenges in mismatch repair: getting over the chromatin hurdle. DNA Repair (Amst) 2014; 19: 48–54. 24767944 10.1016/j.dnarep.2014.03.027 PMC4127414

[124] Namuli KL Slike AN Hollebeke MA , et al. Genomic characterization of Huntington’s disease genetic modifiers informs drug target tractability. Brain Commun 2025; 7: fcae418. 10.1093/braincomms/fcae418 PMC11724427 39801710

[125] Kropach N Shkalim-Zemer V Orenstein N , et al. Novel RRM2B mutation and severe mitochondrial DNA depletion: report of 2 cases and review of the literature. Neuropediatrics 2017; 48: 456–462. 28482374 10.1055/s-0037-1601867

[126] Finsterer J Zarrouk-Mahjoub S . Phenotypic and genotypic heterogeneity of RRM2B variants. Neuropediatrics 2018; 49: 231–237. 29241262 10.1055/s-0037-1609039

[127] Lim AZ McFarland R Taylor RW , et al. Mitochondrial DNA maintenance defects. In: Adam MP Feldman J Mirzaa GM (eds) GeneReviews. Seattle, WA: University of Washington, 2014, pp.1539–1555. 24741716

[128] Lou M Liu Q Ren G , et al. Physical interaction between human ribonucleotide reductase large subunit and thioredoxin increases colorectal cancer malignancy. J Biol Chem 2017; 292: 9136–9149. 28411237 10.1074/jbc.M117.783365 PMC5454097

[129] Martires LCM Ahronian LG Pratt CB , et al. LIG1 Is a synthetic lethal target in BRCA1 mutant cancers. Mol Cancer Ther 2025; 24: 618–627. 39868490 10.1158/1535-7163.MCT-24-0598 PMC11962389

[130] Nakano K Bálint E Ashcroft M , et al. A ribonucleotide reductase gene is a transcriptional target of p53 and p73. Oncogene 2000; 19: 4283–4289. 10980602 10.1038/sj.onc.1203774

[131] Elfar GA Aning O Ngai TW , et al. p53-dependent crosstalk between DNA replication integrity and redox metabolism mediated through a NRF2-PARP1 axis. Nucleic Acids Res 2024; 52: 12351–12377. 39315696 10.1093/nar/gkae811 PMC11551750

[132] Kuo M-L Sy AJ Xue L , et al. RRM2B Suppresses activation of the oxidative stress pathway and is up-regulated by p53 during senescence. Sci Rep 2012; 2: 822. 23139867 10.1038/srep00822 PMC3492868

[133] Lynch DR Chin MP Boesch S , et al. Efficacy of Omaveloxolone in Friedreich’s ataxia: delayed-start analysis of the MOXIe extension. Mov Disord 2023; 38: 313–320. 36444905 10.1002/mds.29286

[134] Ibañez K Jadhav B Zanovello M , et al. Increased frequency of repeat expansion mutations across different populations. Nat Med 2024; 30: 3357–3368. 39354197 10.1038/s41591-024-03190-5 PMC11564083

